# Semaphorin3A-Inhibitor Ameliorates Doxorubicin-Induced Podocyte Injury

**DOI:** 10.3390/ijms21114099

**Published:** 2020-06-08

**Authors:** Yizhen Sang, Kenji Tsuji, Akiko Inoue-Torii, Kazuhiko Fukushima, Shinji Kitamura, Jun Wada

**Affiliations:** Department of Nephrology, Rheumatology, Endocrinology and Metabolism, Graduate School of Medicine, Dentistry and Pharmaceutical Sciences, Okayama University, Okayama 700-8558, Japan; yizhensang@outlook.com (Y.S.); gmd422036@s.okayama-u.ac.jp (K.T.); elle_stage@hotmail.com (A.I.-T.); p7927m3x@s.okayama-u.ac.jp (K.F.); junwada@okayama-u.ac.jp (J.W.)

**Keywords:** semaphorin3A, podocyte, proteinuria, apoptosis, c-Jun N-terminal kinase

## Abstract

Podocyte injury is an independent risk factor for the progression of renal diseases. Semaphorin3A (SEMA3A), expressed in podocytes and tubular cells in the mammalian adult kidneys, has been reported to regulate diverse biological functions and be associated with renal diseases. Here, we investigated pathological roles of SEMA3A signaling on podocyte injury using a doxorubicin (Dox)-induced mouse model and examined the therapeutic effect of SEMA3A-inhibitor (SEMA3A-I). We demonstrated that Dox caused massive albuminuria and podocyte apoptosis as well as an increase of SEMA3A expression in podocytes, all of which were ameliorated with SEMA3A-I treatment. In addition, c-Jun N-terminal kinase (JNK), known as a downstream of SEMA3A signaling, was activated in Dox-injected mouse podocytes while SEMA3A-I treatment partially blocked the activation. In vitro, SEMA3A-I protected against Dox-induced podocyte apoptosis and recombinant SEMA3A caused podocyte apoptosis with activation of JNK signaling. JNK inhibitor, SP600125, attenuated SEMA3A-induced podocyte apoptosis, indicating that the JNK pathway would be involved in SEMA3A-induced podocyte apoptosis. Furthermore, the analysis of human data revealed a positive correlation between levels of urinary SEMA3A and protein, suggesting that SEMA3A is associated with podocyte injury. In conclusion, SEMA3A has essential roles in podocyte injury and it would be the therapeutic target for protecting from podocyte injury.

## 1. Introduction

The renal filtration barrier consists of three layers: endothelial cells, glomerular basement membrane (GBM), and podocytes [1]. Podocytes are terminally differentiated cells that line out of the GBM and act as the final barrier of protein loss. Therefore, podocyte injury is associated with the onset of proteinuria [2]. Proteinuria is a hallmark of glomerular disease which leads to chronic kidney disease (CKD) [3]. Indeed, proteinuria has been reported to be an independent risk factor for the progression to end-stage renal diseases in CKD patients [4,5]. Therefore, halting podocyte injury would protect from the progression of renal diseases.

The semaphorin family is a large group of proteins which are grouped into 5 classes (class 3 to 7) in mammals. Class 3 semaphorins are secreted proteins while the others are transmembrane (class 4, 5 and 6) or glycosylphosphatidylinositol-linked proteins (class 7) [6]. Semaphorins were recognized as key regulators of various cellular functions in many tissues, including kidney, heart, bone, and neuron [7,8]. Class 3 semaphorins comprise 7 proteins, which are semaphorin 3A–3G (SEMA3A–3G). Among these, SEMA3A has been reported to regulate diverse biological functions, including immune system, angiogenesis, and cell migration [8]. Neuropilin-1 (NRP1) and plexinA have been identified as SEMA3A binding and signaling receptors, respectively. SEMA3A binds to NRP1 with high affinity to assemble a NRP1/plexinA receptor complex and is involved in multiple functions [9].

In human kidneys, SEMA3A is expressed in podocytes, luminal aspect of distal tubules, and collecting ducts, while NRP1 is expressed in peritubular endothelia and podocytes [10]. Accumulating evidence has indicated the role of SEMA3A in renal diseases. For example, SEMA3A expression is increased in various glomerular diseases [7]. It has also been suggested that excess SEMA3A binds to NRP1, leading to activation of plexinA, inducing foot process effacement, endothelial injury, and GBM lamination [8,11]. We previously verified that urinary SEMA3A levels correlate with minimal change nephrotic syndrome (MCNS) activity [12], suggesting that the increased SEMA3A expression may be associated with proteinuric kidney diseases. However, the pathophysiological roles of SEMA3A in podocyte injury are unclear.

In the present study, we investigated the role of SEMA3A in renal injury by using a Doxorubicin (Dox)-induced podocytopathy mouse model, an experimental model of focal segmental glomerulosclerosis with time-dependent podocyte apoptosis [13], and examined the therapeutic effect of a selective SEMA3A inhibitor (SM-345431: vinaxanthone, SEMA3A-I) [14,15,16].

## 2. Results

### 2.1. Podocyte SEMA3A Expression is Increased in a Dox-Induced Mouse Model

**Figure 1:**
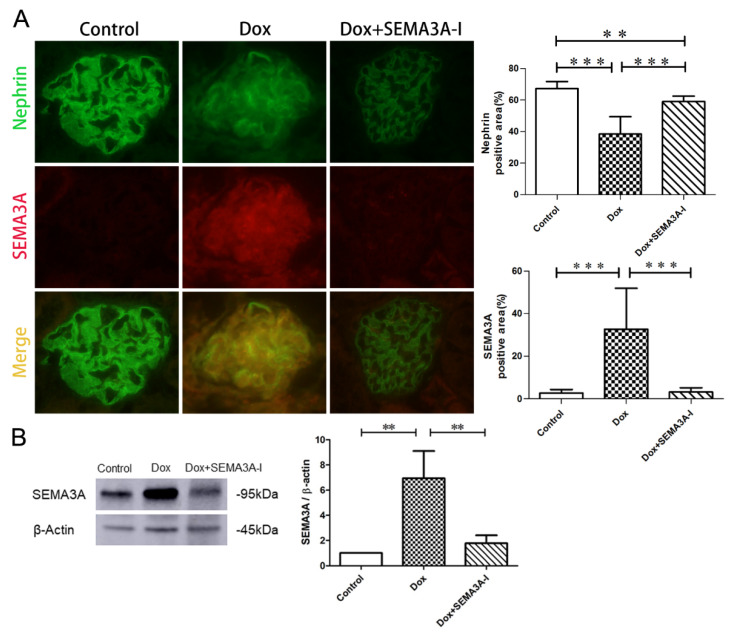
The expression of Semaphorin3A (SEMA3A) is increased in podocytes from Doxorubicin (Dox)-induced renal injury mice. (**A**) Dual immunofluorescence staining of SEMA3A (red) and nephrin (green) in mouse glomeruli from the control, Dox, and Dox + SEMA3A-inhibitor (SEMA3A-I) groups at the time point of 2 weeks after Dox injection, showing the increase of SEMA3A expression and decrease of nephrin expression in Dox-treated podocytes. Representative images are shown. Original magnification, ×400. The nephrin and SEMA3A-positive area/glomeruli (%) are shown in the graphs. (**B**) Western blotting analysis of SEMA3A and β-actin in the control, Dox, and Dox + SEMA3A-I groups at the time point of 2 weeks after Dox injection, showing the increase of SEMA3A in the Dox group. Densitometric analysis was performed to quantify the Western blotting results. Data are shown from three independent experiments. ** *p* < 0.01, *** *p* < 0.001.

Since excess SEMA3A has been reported to be associated with various glomerular diseases [7], we applied the Dox-induced podocytopathy mouse model and examined the change of SEMA3A expression. SEMA3A expression in podocytes was dramatically increased in the Dox group compared to the control group (Figure 1A). In addition, no significant increase in SEMA3A expression was detected in the Dox + SEMA3A-I group compared to the control group (Figure 1A). These results were consistent with the results obtained by Western blotting (Figure 1B). Furthermore, nephrin expression was significantly decreased in the Dox group compared to the control group, which was partially ameliorated in the Dox + SEMA3A-I group (Figure 1A), suggesting that SEMA3A-I might protect from Dox-induced podocyte injury.

### 2.2. SEMA3A-Inhibitor Protected from Dox-Induced Podocyte Injury

**Figure 2:**
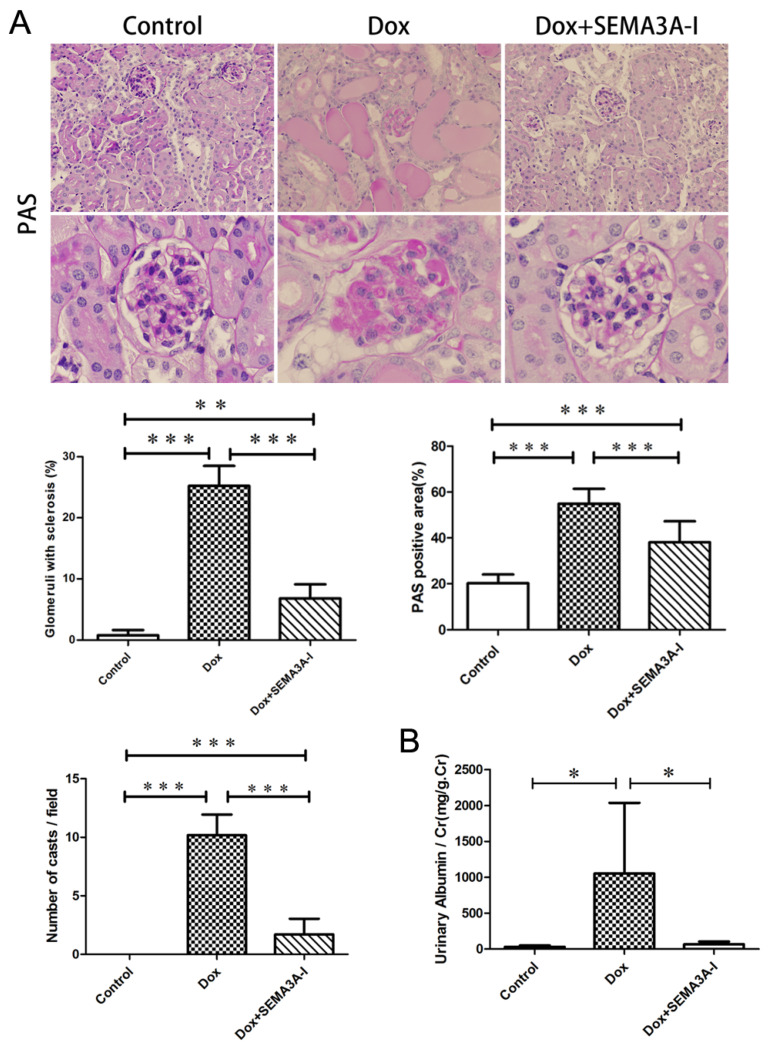
Inhibition of SEMA3A protected against Doxorubicin-induced podocyte injury and albuminuria. (**A**) Histological manifestations are determined by periodic acid-Schiff (PAS) staining to assess the glomerular injury in the control, Dox, and Dox + SEMA3A-I groups at the time point of 2 weeks after Dox injection. Glomerular structure and podocytes were severely damaged with tubular casts in the Dox group compared to the control and Dox + SEMA3A-I groups. Representative images are shown. Original magnification, ×200 (upper panel), ×400 (lower panel). The PAS-positive area/glomeruli (%), the ratio of glomeruli with sclerosis (%), and the number of tubular casts/200× fields are shown in the graphs. ** *p* < 0.01, *** *p < 0.001*. (**B**) Mouse urinary albumin/creatinine ratio in the control, Dox, and Dox + SEMA3A-I groups at the time point of 2 weeks after Dox injection, showing higher levels of urinary albumin in the Dox group compared to the control and Dox + SEMA3A-I groups. Analysis was conducted by the Wilcoxon test. * *p* < 0.05.

To determine the effect of SEMA3A-I in Dox-induced podocyte injury, we examined these mouse kidneys histopathologically. Periodic acid-Schiff (PAS) staining images revealed that podocytes were severely damaged in the Dox group compared to that of the control group (Figure 2A). Numerous tubular casts were also detected in the Dox group (Figure 2A). On the other hand, these podocytopathy and tubular casts were fewer in the Dox + SEMA3A-I group compared to the Dox group (Figure 2A). In addition, urinary albumin levels were significantly increased in the Dox group compared to the control group, while there was no significant difference between the Dox + SEMA3A-I group and the control group (Figure 2B). These results indicated that SEMA3A-I protected from Dox-induced podocyte injury.

### 2.3. SEMA3A-Inhibitor Protected from Dox-Induced Podocyte Apoptosis

**Figure 3:**
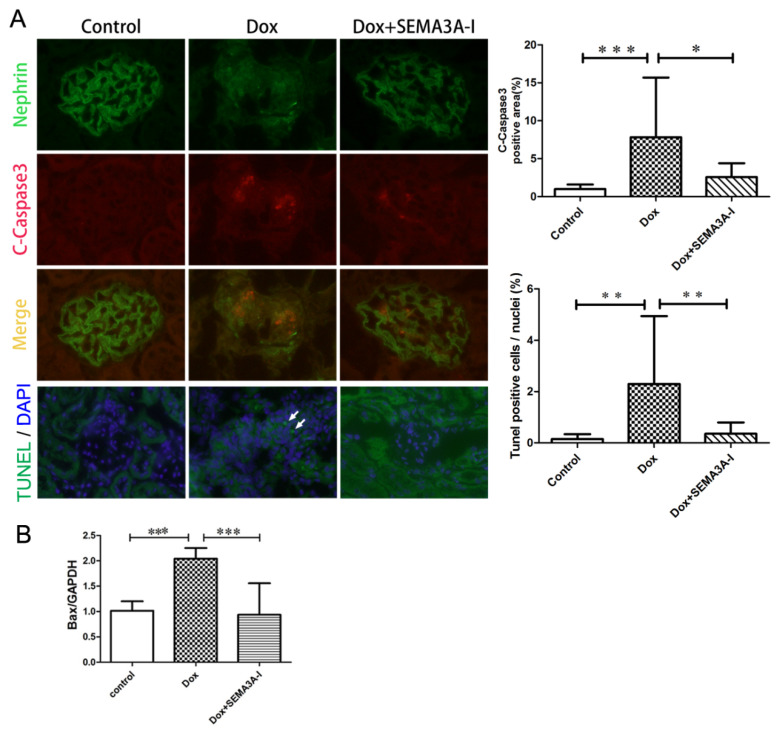
SEMA3A-inhibitor protected from Doxorubicin-induced podocyte apoptosis. (**A**) Dual immunofluorescence staining of cleaved-caspase3 (C-Caspase3) (red) and nephrin (green) in mouse glomeruli from the control, Dox, and Dox + SEMA3A-I groups at the time point of 2 weeks after Dox injection, showing the increase of C-Caspase3-positive podocytes in the Dox group, and fewer C-Caspase3-positive cells in the Dox + SEMA3A-I group. Images of immunofluorescent staining of TdT-mediated dUTP Nick-End Labeling (TUNEL, green) and 4′,6′-diamidino-2-phenylindole (DAPI, blue) in the control, Dox, and Dox + SEMA3A-I groups (Lowest panel) show that TUNEL-positive cells were detected in the Dox group (white arrows), while almost no TUNEL-positive cells were detectable in the control and Dox + SEMA3A-I groups. Representative images are shown. Original magnification, ×400 (C-Caspase3 and nephrin) and x200 (TUNEL). C-Caspase3-positive area/glomeruli (%) and TUNEL-positive cells/nuclei (%) are shown in the graphs. (**B**) Reverse transcription-quantitative polymerase chain reaction (RT-qPCR) analysis of B cell lymphoma2-associated x-protein (Bax)/Glyceraldehyde 3-phosphate dehydrogenase (GAPDH) ratio in the control, Dox, and Dox + SEMA3A-I groups at the time point of 2 weeks after Dox injection, showing the increase of Bax mRNA level in the Dox group. * *p* *<* 0.05, ** *p* *<* 0.01, *** *p* < 0.001.

To explore the mechanism by which SEMA3A-I protected from Dox-induced podocytopathy, we proceeded to further analysis. Since a previous report indicated that inhibition of SEMA3A ameliorated lipopolysaccharide (LPS)-induced kidney injury via inhibition of apoptosis [17], we examined the apoptosis by cleaved-Caspase3 (C-Caspase3) staining and TdT-mediated dUTP Nick-End Labeling (TUNEL) staining. The expression of C-Caspase3 was higher in the Dox group compared to the control and the Dox + SEMA3A-I groups (Figure 3A). In addition, TUNEL staining analysis revealed higher TUNEL-positive cells in the kidneys in the Dox group, while there were almost no TUNEL-positive cells in the control group (Figure 3A). Importantly, we rarely detected TUNEL-positive cells in the Dox + SEMA3A-I group, indicating that SEMA3A-I protected from Dox-induced podocyte apoptosis. In addition, reverse transcription-quantitative polymerase chain reaction (RT-qPCR) analysis revealed an increase of pro-apoptotic marker B cell lymphoma2-associated x-protein (Bax) in the Dox group compared to the control and Dox + SEMA3A-I groups, confirming the inhibition of apoptosis with SEMA3A-I treatment (Figure 3B).

### 2.4. SEMA3A-Inhibitor Reduced Dox-Induced JNK/c-Jun Signaling

**Figure 4:**
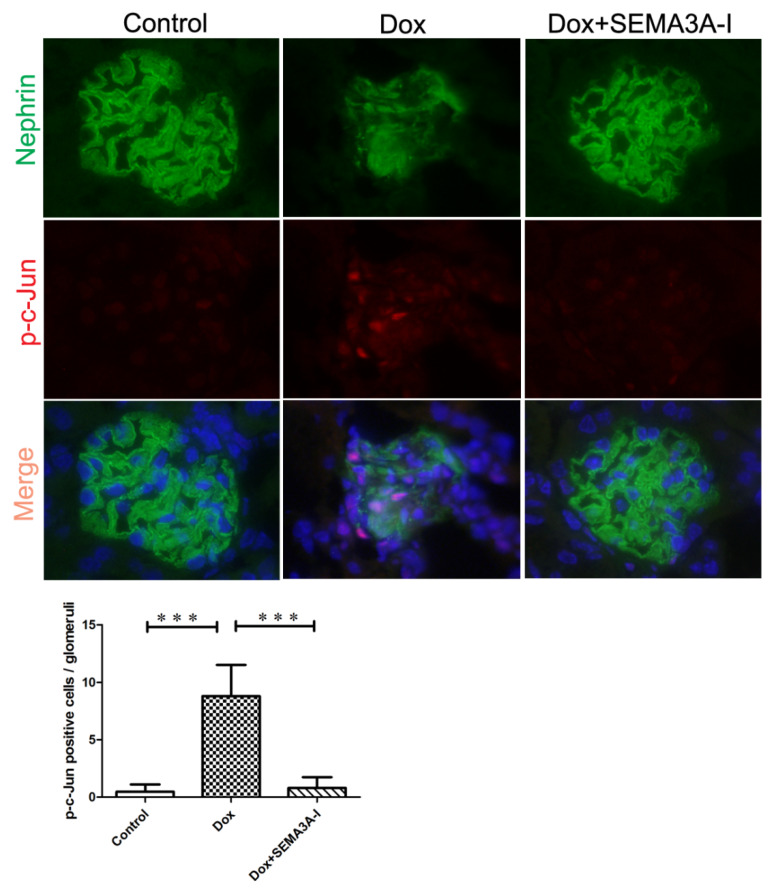
SEMA3A-inhibitor attenuated Doxorubicin-induced c-Jun N-terminal kinase (JNK)/c-Jun signaling. Representative images of dual immunofluorescence staining of nephrin (green), p-c-Jun (red), and DAPI (blue) in glomeruli from the control, Dox, and Dox + SEMA3A-I groups at the time point of 2 weeks after Dox injection, showing the increased p-c-Jun-positive cells in glomeruli in the Dox group, while there were few p-c-Jun-positive cells in the Dox + SEMA3A-I group. Original magnification, ×400. p-c-Jun-positive cells/glomeruli was shown in the graph. *** *p* *<* 0.001.

The c-Jun N-terminal kinase (JNK) pathway is one of the important signaling cascades of the mitogen-activated protein kinase (MAPK) pathway, which functions in various cellular processes including proliferation, differentiation, migration, and apoptosis [18]. SEMA3A has been reported to activate the JNK pathway in neurons [19]. We therefore investigated whether the JNK/c-Jun pathway was involved in Dox-induced renal injury and/or SEMA3A-NRP1 signaling. Phospho-c-Jun (p-c-Jun)-positive cells in glomeruli by immunofluorescence staining were increased in the Dox group compared to the lack of p-c-Jun-positive cells in the control group and fewer p-c-Jun-positive cells in the Dox + SEMA3A-I group (Figure 4), suggesting that Dox-induced cell apoptosis and renal injury are largely dependent on the JNK/c-Jun signaling pathway.

### 2.5. SEMA3A Caused Podocyte Apoptosis In Vitro

**Figure 5:**
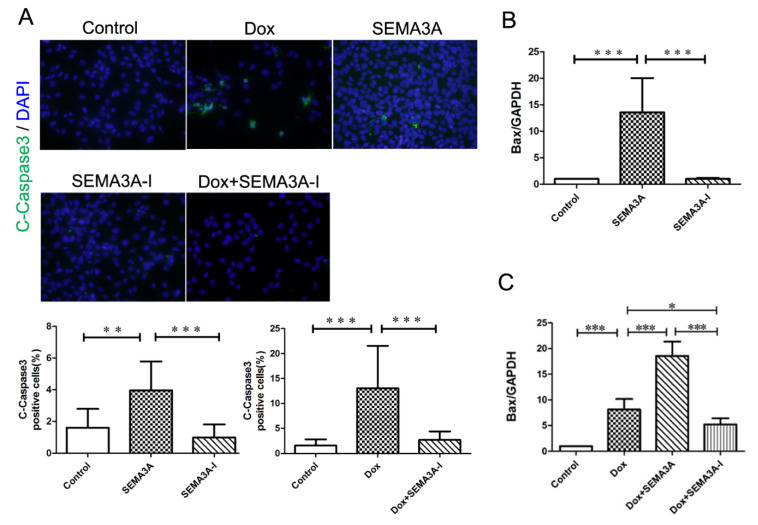
SEMA3A caused podocyte apoptosis while SEMA3A-inhibitor protected from podocyte apoptosis. (**A**) Representative immunofluorescence staining images of cleaved-caspase3 (C-Caspase3) (green) and DAPI (blue) in podocytes with or without Dox (0.5 μg/mL), SEMA3A (50 ng/mL), SEMA3A-I (0.5 μM), and Dox (0.5 μg/mL) + SEMA3A-I (0.5 μM). Original magnification, ×200. C-Caspase3-positive cells/nuclei (%) in 200× field are shown in the graphs. (**B**,**C**) RT-qPCR analysis of Bax and GAPDH mRNA with or without SEMA3A (50 ng/mL), SEMA3A-I (0.5 μM), Dox (0.5 μg/mL), Dox (0.5 μg/mL) + SEMA3A (50 ng/mL), and Dox (0.5 μg/mL) + SEMA3A-I (0.5 μM), showing that the mRNA level of Bax is increased with SEMA3A or Dox treatment, and is decreased with the SEMA3A-I treatment. * *p* < 0.05, ** *p* < 0.01, *** *p* < 0.001.

We further conducted in vitro analysis using immortalized mouse podocytes. The immunofluorescence staining with C-Caspase3 and 4′,6′-diamidino-2-phenylindole (DAPI) revealed that SEMA3A as well as Dox treatment resulted in the increase of C-Caspase3-positive cells (Figure 5A), confirming that SEMA3A may cause podocyte apoptosis. Importantly, less C-Caspase3-positive cells were detected in podocytes with Dox + SEMA3A-I treatment compared to podocytes with Dox treatment (Figure 5A), indicating that SEMA3A-I might protect from Dox-induced podocyte apoptosis. We also conducted RT-qPCR analysis, revealing that Dox treatment as well as SEMA3A treatment in podocytes significantly increased Bax mRNA expression (Figure 5B,C). In addition, SEMA3A-I partially blocked the increased expression of Bax mRNA caused by Dox (Figure 5C). These results indicated that SEMA3A may cause podocyte apoptosis, while SEMA3A-I may protect from Dox-induced podocyte apoptosis.

### 2.6. JNK-Inhibitor Attenuated SEMA3A-Induced Podocyte Apoptosis

**Figure 6:**
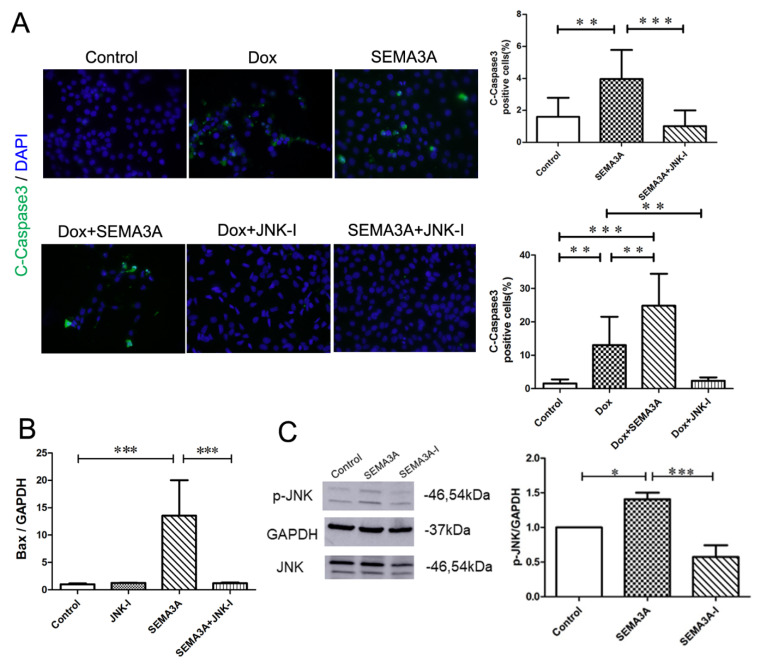
JNK-inhibitor blocked SEMA3A-induced podocyte apoptosis. (**A**) Immunofluorescence staining of cleaved-caspase 3 (C-Caspase3) (green) and DAPI (blue) in podocytes with or without Dox (0.5 μg/mL), SEMA3A (50 ng/mL), Dox (0.5 μg/mL) + SEMA3A (50 ng/mL), Dox (0.5 μg/mL) + JNK-inhibitor (JNK-I) (10 μM), SEMA3A (50 ng/mL) + JNK-I (10 μM), showing that JNK-I prevented from Dox- and SEMA3A-induced podocyte apoptosis. Representative images are shown. Original magnification, ×200. C-Caspase3-positive cells/nuclei (%) in 200× fields are shown in the graphs. (**B**) RT-qPCR analysis of Bax and GAPDH mRNA with or without SEMA3A (50 ng/mL), JNK-I (10 μM), and SEMA3A (50 ng/mL) + JNK-I (10 μM), showing that mRNA level of Bax is increased with SEMA3A treatment, and is decreased with the JNK-I treatment. (**C**) Western blotting analysis of p-JNK, JNK, and GAPDH with or without SEMA3A (50 ng/mL) or SEMA3A-I (0.5 μM), showing that SEMA3A upregulated the expression of p-JNK. Densitometric analysis was performed to quantify the Western blotting results. Data are shown from three independent experiments. * *p* < 0.05, ** *p* <0.01, *** *p* < 0.001.

To examine whether the JNK/c-Jun pathway is involved in SEMA3A-induced podocyte apoptosis, we used the JNK-inhibitor, SP600125 (JNK-I). JNK-I reduced C-Caspase3-positive apoptotic podocytes induced by Dox or SEMA3A (Figure 6A), demonstrating that JNK signaling might be involved in the SEMA3A-related podocyte apoptosis. In addition, RT-qPCR analysis revealed that JNK-I attenuated increased expression of Bax mRNA caused by SEMA3A (Figure 6B). We also confirmed the increase of JNK signaling in podocyte with SEMA3A treatment by Western blotting (Figure 6C). Taken together, these results indicated that SEMA3A induced podocyte apoptosis through the JNK/c-Jun signaling pathway.

### 2.7. Positive Correlation between Urinary SEMA3A and Proteinuria is Present in Human Samples

**Figure 7:**
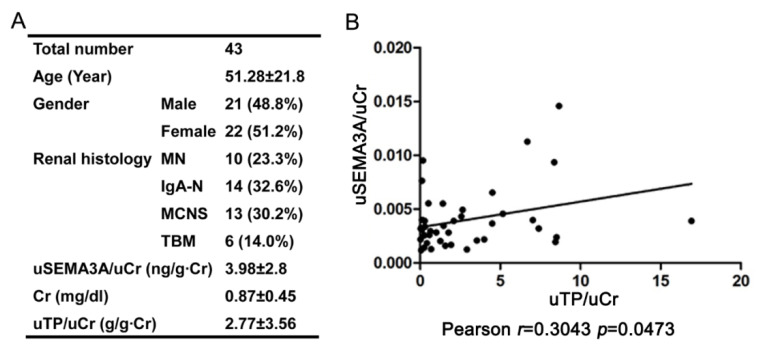
Positive correlation is present between urinary SEMA3A and proteinuria in human urine samples from biopsied patients. (**A**) Characteristics of the patients. Values are mean ± standard deviation (SD). Percentages are reported for categorical variables. Cr, serum creatinine; uCr, urinary creatinine; uTP, urinary total protein; uSEMA3A, urinary SEMA3A; IgA-N: IgA nephritis; MN, membranous nephropathy; TBM, thin basement membrane disease; MCNS, minimal change nephrotic syndrome. (**B**) The relationship between the uSEMA3A/uCr ratio (ng/g Cr) and uTP/uCr ratio (g/g∙Cr) are shown. uSEMA3A level was positively correlated with urine protein (*n* = 43). *r* = 0.3043, *p* = 0.0473.

We further explored the association between urinary SEMA3A and urinary protein using urine samples from biopsied patients (*n* = 43). The characteristics of these patients were shown in Figure 7A with the patients of IgA nephritis (IgA-N), membranous nephropathy (MN), thin basement membrane disease (TBM), and MCNS. The statistical analysis revealed the positive correlation between urinary SEMA3A level and proteinuria (Figure 7B), suggesting that increased SEMA3A expression is associated with podocyte injury and proteinuria.

## 3. Discussion

SEMA3A has been reported to play important roles in multiple aspects of renal diseases [7]. For example, urinary SEMA3A has been shown to be an early, predictive biomarker of acute kidney injury (AKI) as well as later-onset AKI and progression of AKI [20,21]. Previously, we reported that urinary SEMA3A levels in MCNS, IgA-N, and MN groups were higher than in the control group, and that urinary SEMA3A might be an indicator for remission of MCNS patients [12]. Consistent with these reports, Aggarwal et al. reported that excess SEMA3A might promote diabetic nodular glomerulosclerosis, massive proteinuria, and renal failure in diabetic nephropathy mice [22]. Likewise, Mohamed et al. reported increased levels of urinary SEMA3A in diabetic mice and human diabetic patients with nephropathy [23]. In addition, it has also been reported that SEMA3A can be a predictive biomarker for ankylosing spondylitis [24] and systemic lupus erythematosus [25], suggesting the effect of SEMA3A signaling on the regulation of the immune system. These findings suggest that SEMA3A has common effects on the podocyte injury and subsequent progression of renal injury in renal diseases. Our results indicated the increased expression of podocyte SEMA3A in Dox-induced mouse kidneys, and SEMA3A-I protected from Dox-induced renal injury by lowering albuminuria and podocyte injury, demonstrating that SEMA3A signaling does have the association with podocyte injury and targeting the SEMA3A–NRP1 axis with SEMA3A-I would be a therapeutic option to treat renal injury. Interestingly, SEMA3A-I treatment also inhibited Dox-induced SEMA3A expression in podocytes, which seemed somehow strange considering the effect of SEMA3A-I to block the binding of SEMA3A–NRP1. We assumed that SEMA3A-I attenuated Dox-induced podocytopathy, which resulted in the reduction of SEMA3A from injured podocytes. In addition, there was the positive correlation between urinary SEMA3A level and proteinuria in a human study, reinforcing our conclusion.

On the point of SEMA3A inhibition, several chemicals have been reported. For example, Tian et al. have applied (-)-Epigallocatechin-3-gallate (EGCG) as a SEMA3A inhibitor, which is the major polyphenol constituent from green tea, in LPS-induced AKI [17,26,27]. They indicated the increase in tubular SEMA3A expression with LPS treatment, and EGCG suppressed LPS-induced cell apoptosis and inflammation through the regulation of JNK and Rac1/NF-κB p65 signaling [17]. In addition, Kumagai et al. identified a novel, highly selective SEMA3A inhibitor (SM-345431, vinaxanthone) [14]. SM-345431 was isolated from the cultured broth of a fungus *Penicillium sp.* and interacts with SEMA3A directly and inhibits the binding of SEMA3A to NRP1 with the same physicochemical properties as SM-216289 (xanthofulvin), but develops a higher pharmaceutical quality [14,15,16]. Indeed, SM-345431 has been shown to enhance regenerative response and functional recovery of the injured spinal cord [28]. It is also reported that SM-345431 accelerated peripheral nerve regeneration and sensitivity in a murine corneal transplantation model [29]. In this study, we demonstrated that SM-345431 protected from Dox-induced podocyte injury through an anti-apoptosis mechanism.

Among broad biological functions of SEMA3A, several reports indicated important roles of SEMA3A signaling on the regulation of cell apoptosis through the SEMA3A–NRP1/JNK axis [17,30]. The JNK/c-Jun pathway belongs to MAPK signaling, which can be activated by diverse stimulus, including reactive oxygen stress, inflammatory cytokines, and mechanical stress [31,32]. The JNK/c-Jun pathway has been shown to promote apoptosis in a variety of cell types [32]. In addition to the JNK pathway, various pathways, including Janus kinase/signal transducers and activators of transcription (JAK/STAT), protein kinase B (Akt) and other MAPK pathways of extracellular signal-regulated kinase (ERK) and p38, play critical roles in the cell apoptosis/survival paradigm. Wen et al. reported that macrophages, inhibiting SEMA3A signaling by knockout of plexinA4, reduced JNK phosphorylation, but no change was observed in phosphorylation of ERK1/2, p38, STAT1, and Akt under the stimulation [33], suggesting that SEMA3A signaling might specifically regulate JNK signaling. Therefore, we focused on the SEMA3A–JNK axis in the present study. On the other hand, it is also reported that SEMA3A signaling regulates dendritic development through the activation of the Akt pathway [34]. In addition, Guan et al. reported that SEMA3A might decrease Akt phosphorylation and induce apoptosis in cultured podocytes [35], indicating the possibility that SEMA3A might also regulate pathways other than JNK signaling. While SEMA3A-induced podocyte apoptosis was induced through the regulation of JNK signaling in the present study, which demonstrated the involvement of the JNK pathway in SEMA3A signaling, at least to some extent, further analysis is required to elucidate the detailed mechanisms by which SEMA3A signaling regulates the cell apoptosis/survival paradigm.

On the point of the therapeutic target for the MAPK pathway, there are several candidates reported to protect against podocyte injury. For example, Liu et al. reported that activation of ERK signaling as well as the JNK pathway was observed in a rat puromycin aminonucleoside (PAN) nephropathy model, and that treatment with U0126, an inhibitor of ERK, suppressed podocyte apoptosis caused by PAN [36]. Yu et al. reported that transforming growth factor beta 1 (TGF-β1)-induced podocyte injury was ameliorated with U0126 treatment through the inhibition of the increment of transient receptor potential cation channel 6 (TRPC6) protein [37]. Lei et al. reported that mammalian target of rapamycin (mTOR) activation is associated with endoplasmic reticulum (ER) stress and apoptosis in high-glucose-treated podocyte, which was ameliorated with U0126 treatment [38]. Taken together, targeting ERK pathway might be a potential target for podocyte injury. In addition to the ERK pathway, involvement of the p38 pathway under podocyte injury is also reported. Koshikawa et al. reported the activation of the p38 MAPK and ERK pathways in rodent PAN and Dox nephropathy models, and that the treatment with FR167653, an inhibitor of p38 MAPK, completely blocked the increase of proteinuria caused by PAN or Dox [39]. Pengal et al. applied another inhibitor of p38 MAPK, SB203580, which reduced PAN-induced podocytopathy and actin cytoskeletal disruption [40], indicating the therapeutic potential of p38 inhibition under podocytopathy. Furthermore, the blockade of the JNK/c-Jun pathway has been shown to suppress renal injury in several disease models and has therapeutic value [18,41,42]. However, clinical trials evaluating JNK inhibitors in human fibrotic disorders showed side effects of liver toxicity [43]. Thus, other therapeutic strategies are needed to reduce the renal injury via the JNK/c-Jun pathway. On this point, apoptosis signal-regulating kinase 1 (ASK1), the member of the mitogen-activated protein kinase kinase kinase (MAPKKK) family, is another candidate to regulate the JNK/c-Jun pathway [44]. Accumulating evidence revealed that ASK1 activation accelerates renal injury through the activation of p38 and JNK cascades in rodent models of kidney injury, including ischemia/reperfusion-induced AKI, unilateral ureteric obstruction, and diabetic nephropathy [45,46,47], and that treatment with GS-444217, an inhibitor of ASK1, limited the loss of podocytes most likely through the anti-apoptosis pathway in a diabetic kidney disease mouse model, indicating ASK1 as an important target for podocyte injury [48]. In the present study, we demonstrated that SEMA3A-I may decrease podocyte apoptosis through the suppression of the JNK/c-Jun pathway, indicating that SEMA3A might be another candidate to target JNK pathway for podocyte protection.

Another important point to discuss is the timing of the treatment with SEMA3A-I or JNK-I. In our in vitro experiment, we added these inhibitors and/or Dox or SEMA3A at the same time. To examine whether these inhibitors might revert the podocyte apoptosis caused by Dox or SEMA3A, we might make the time lag to add these inhibitors after Dox or SEMA3A treatment. We assume that these inhibitors might not revert the podocyte apoptosis after the apoptosis signaling cascade proceeds over the SEMA3A or JNK signaling. In such situation, we would propose to use these inhibitors before or under the podocyte injury, not after the podocytopathy has been established. Further study is still required to understand the timing at which these potential inhibitors work and how SEMA3A-I functions on the JNK/c-Jun pathway in a Dox-induced podocytopathy mouse model.

In conclusion, our results demonstrated that SEMA3A-I treatment protected from Dox-induced podocytopathy by inhibiting podocyte apoptosis through the regulation of the JNK pathway. It would be the therapeutic target for preventing podocyte injury.

## 4. Materials and Methods

### 4.1. Animal Experimental Design

The experimental protocol was approved by the Animal Ethics Review Committee of the Okayama University Graduate School of Medicine, Dentistry and Pharmaceutical Sciences (OKU-2013187, approved on 2013/05/20). The studies were performed on 10-week-old wild-type BALB/c mice obtained from CLEA (CLEA Japan, Tokyo, JAPAN). The animals had free access to tap water and standard mouse chow. All the experiments were performed in accordance with relevant guidelines and regulations.

### 4.2. Mouse Model of Doxorubicin-Induced Podocytopathy

Mice were randomly divided into three groups (*n* = 5 each group): control, Dox, and Dox + SEMA3A-I. The control group received 100 µl saline (Otsuka Pharmaceutical Factory, Inc., Tokushima, Japan) injection by tail vein. Mice in the Dox group were injected with Dox (Sigma-Aldrich, St. Louis, MO, USA), 10 mg/kg body weight, by tail vein. In the Dox + SEMA3A-I group, mice were injected with Dox, 10 mg/kg body weight, by tail vein, followed by a daily intraperitoneal injection of 20 µg SEMA3A-I (SM-345431, provided by Sumitomo Dainippon Pharma Co, Ltd., Osaka, Japan [14]). All the mice were euthanized and individual 24-h urine samples, blood samples, and kidney tissues were collected at the time point of 2 weeks after the Dox injection.

### 4.3. Histological Examination

Collected kidney tissues were fixed with 10% buffered formalin (Nacalai Tesque, Kyoto, Japan) and were embedded in paraffin. Sections (4 µm thick) were stained with PAS staining for light microscopy analysis. The PAS-positive area was evaluated in 15 randomly selected glomeruli at 400× magnification using the ImageJ software (available at http://rsbweb.nih.gov/ij/index.html; National Institutes of Health). The ratio of glomeruli with sclerosis was evaluated in 100 randomly selected glomeruli. The number of tubular casts was counted in 15 randomly selected fields at 200× magnification.

### 4.4. Immunohistochemical Staining

Immunohistochemical staining was conducted as previously reported [49]. Briefly, frozen kidney tissues were subjected to cryosectioning (4 µm thick) and immunofluorescence staining with the primary antibodies (Table 1) (Abcam, Cambridge, UK; Progen, Heidelberg, Germany; Cell Signaling Technology, Danvers, MA, USA), corresponding secondary antibodies from Invitrogen (Waltham, MA, USA) (Alexa Fluor 594 goat anti-rabbit antibody, Alexa Fluor 488 goat anti-guinea pig antibody, Alexa Fluor 488 donkey anti-rabbit antibody), and DAPI (Roche Diagnostics GmbH, Mannheim, Germany). The number of p-c-Jun-positive cells was counted in 15 randomly selected glomeruli at 400× magnification. Nephrin-, SEMA3A-, and the C-Caspase3-positive areas were evaluated in 15 randomly selected glomeruli at 400× magnification using the ImageJ software. For in vitro staining, cells were plated on chamber slides (ThermoFisher Scientific, Waltham, MA, USA), allowed to grow up to 90% confluence, and then they were subjected to further experiments. At the end of the experiments, cells were fixed with 4% paraformaldehyde (PFA) (Nacalai Tesque, Kyoto, Japan) in phosphate-buffered saline (PBS) (ThermoFisher Scientific, Waltham, MA, USA) for 20 min, and were permeabilized with 0.01% Triton X-100 (ThermoFisher Scientific, Waltham, MA, USA) in PBS for 4 min, followed by the immunofluorescence staining. C-Caspase3-positive cells were evaluated in 10 randomly selected fields at 200× magnification using the ImageJ software. All images were recorded on an FSX-100 microscope (Olympus Corporation, Tokyo, Japan).

### 4.5. TUNEL Staining

The degree of podocyte apoptosis was assessed by using the terminal deoxynucleotidyl transferase-mediated TUNEL technique (TB235, Promega, Fitchburg, WI, USA), according to the manufacturer’s instruction, and were counterstained with DAPI. The number of TUNEL-positive cells/nuclei was counted in 15 randomly selected fields at 200× magnification. The images were recorded on an FSX-100 microscope (Olympus, Tokyo, Japan).

### 4.6. Cell Culture

Conditionally immortalized mouse podocytes, provided from Dr. Peter Mundel [50], were cultured on collagen type I (BD Biosciences, San Jose, CA, USA)-coated dishes and maintained at the permissive condition of 33 °C in Roswell Park Memorial Institute (RPMI) 1640 medium (ThermoFisher Scientific), supplemented with 10% fetal bovine serum (FBS) (ThermoFisher Scientific, Waltham, MA, USA), 1% penicillin and streptomycin (ThermoFisher Scientific, Waltham, MA, USA), and 200 U/mL γ-interferon (Sigma-Aldrich, St. Louis, MO, USA). The podocytes were transferred to a non-permissive condition of 37 °C without γ-interferon for cell differentiation for about 7–14 days and were used for the further experiment. For cell treatment, podocytes were seeded on 6-well pre-coated plates or a 10 cm dishes. Cell starvation was conducted for 24 h (starvation medium supplemented with 0.5% FBS, 1% penicillin and streptomycin) and cells were treated with Dox, 0.5 µg/mL (D1515; Sigma-Aldrich, St. Louis, MO, USA), SEMA3A, 50 ng/mL (5926-S3/CF; R&D Systems, Minneapolis, NE, USA), SEMA3A-I, 0.5 µM (SM-345431), and JNK-I, 10 µM (SP600125, Santa Cruz Biotechnology, Dallas, TX, USA) for 24 h. The immunofluorescence staining, RT-qPCR, and Western blotting were then conducted.

### 4.7. Western Blotting

Sodium Dodecyl Sulfate Polyacrylamide Gel Electrophoresis (SDS-PAGE) and Western blotting were performed as reported previously [49]. Briefly, protein samples were harvested using radio immunoprecipitation assay (RIPA) Lysis and Extraction Buffer (ThermoFisher Scientific, Waltham, MA, USA) with phosphatase inhibitor cocktail (Abcam, Cambridge, UK) and protease inhibitor cocktail (Promega, Fitchburg, WI, USA). Protein concentrations were determined by using a bicinchoninic acid (BCA) protein Assay Kit (ThermoFisher Scientific, Waltham, MA, USA). The equal amount of proteins (15 μg) were loaded onto SDS-PAGE gels, by using Mini-PROTEAN TGX Precast Gels (Bio Rad Laboratories, Hercules, CA, USA), and blocked with skim milk (Nacalai Tesque, Kyoto, Japan) in Tris-Buffered Saline (TBS) buffer containing 0.1% Tween 20 (TBST) (Sigma-Aldrich, St. Louis, MO, USA). The membranes were incubated with the primary antibodies (Table 1), followed by the secondary antibody (Goat anti-Rabbit Horseradish peroxidase conjugate (Bio Rad Laboratories, Hercules, CA, USA)). Immunoreactive bands were visualized by an enhanced chemiluminescence detection system (GE Healthcare, Chicago, IL, USA) and were analyzed by Amersham Imager 600 (GE Healthcare). Optical density of the bands was quantified by densitometric analysis by using ImageJ (available at http://rsbweb.nih.gov/ij/index.html; National Institutes of Health).

### 4.8. Real-Time Quantitative Polymerase Chain Reaction

Total RNA was extracted from kidney tissues and cultured podocytes by using a RNeasy Plus Micro Kit (QIAGEN GmhH, Hilden, Germany). The RNA concentration was measured by spectrophotometry (Nanodrop 2000, Thermo Scientific, Waltham, MA, USA), and complementary DNA was synthesized through a reverse transcription reaction (ThermoFisher Scientific, Waltham, MA, USA). Quantitative PCR was performed with a TaqMan PCR kit (ThermoFisher Scientific, Waltham, MA, USA) according to the manufacturer’s protocol. The gene expression of Bax was relative to Glyceraldehyde 3-phosphate dehydrogenase (GAPDH) and was determined with the 2^−∆∆Ct^ method. Reactions were performed in triplicate, and each experiment was repeated three times. Primers were purchased from ThermoFisher Scientific (Waltham, MA, USA) for GAPDH (Mn99999915_g1) and Bax (Mn00432051_m1).

### 4.9. Human Study

This study is a retrospective analysis using patients’ urine samples. The study was approved by the institutional review board of the Okayama University Graduate School of Medicine, Dentistry and Pharmaceutical Sciences (approval number, 2063: approved on 2014/07/29). This study was registered with the Clinical Trial Registry of the University Hospital Medical Information Network (UMIN000013422 and UMIN000010140). We studied 43 Japanese patients admitted to the Okayama University Hospital for the investigation of renal biopsies between 2011 and 2016. The patients were 16–90 years of age. Informed consent was obtained from all subjects or, if subjects were under 18, from a parent. All the patients were diagnosed according to the combinations of pathological findings of renal biopsies and clinical findings. All methods were carried out in accordance with relevant guidelines and regulations or the Declaration of Helsinki.

### 4.10. Enzyme-Linked Immunosorbent Assay

Human urinary SEMA3A levels were measured by an enzyme-linked immunosorbent assay kit (MyBioSource, Inc., San Diego, CA, USA), as previously described [12]. The levels of SEMA3A were determined by comparing the optical density (O.D) at 450 nm using a microplate reader (Thermo Scientific, Waltham, MA, USA).

### 4.11. Statistical Analysis

Data were presented as mean ± standard deviation (SD). Statistical significance was defined by one-way analysis of variance (ANOVA) with Tukey’s post-test, if not indicated otherwise. The Pearson correlation was used to determine the correlations between two variables. Analyses were performed using GraphPad Prism5.0 software (GraphPad Software Inc., San Diego, CA, USA) and JMP (SAS Institute Inc., Cary, NC, USA). Significance was defined as *p* < 0.05.

## Figures and Tables

**Table 1:** Summary of antibodies used for immunofluorescence and immunoblotting.

Antigen	Manufacturer	Species	Dilution (IF)	Dilution (IB)
SEMA3A	Abcam	Rabbit	1:100	1:1000
Nephrin	Progen	Pig	1:200	-
C-Caspase3	Cell Signaling	Rabbit	1:300	-
p-c-Jun	Cell Signaling	Rabbit	1:200	-
p-JNK	Cell Signaling	Rabbit	-	1:1000
JNK	Cell Signaling	Rabbit	-	1:1000
β-actin	Abcam	Rabbit	-	1:2000
GAPDH	Cell Signaling	Rabbit	-	1:5000

IF, immunofluorescence; IB, immunoblotting; SEMA3A, Semaphorin3A; C-Caspase3, cleaved-Caspase3; p-c-Jun, phospho-c-Jun; p-JNK, phospho-c-Jun N-terminal kinase; GAPDH, Glyceraldehyde 3-phosphate dehydrogenase.

## Abbreviations

SEMA3A	Semaphorin3A
Dox	doxorubicin
JNK	c-Jun N-terminal kinase
GBM	glomerular basement membrane
CKD	chronic kidney disease
NRP1	Neuropilin-1
MCNS	minimal change nephrotic syndrome
PAS	Periodic acid-Schiff
LPS	lipopolysaccharide
C-Caspase3	cleaved-Caspase3
TUNEL	TdT-mediated dUTP Nick-End Labeling
RT-qPCR	reverse transcription-quantitative polymerase chain reaction
Bax	B cell lymphoma2-associated x-protein
MAPK	mitogen-activated protein kinase
p-c-Jun	Phospho-c-Jun
DAPI	4′,6′-diamidino-2-phenylindole
IgA-N	IgA nephritis
MN	membranous nephropathy
TBM	thin basement membrane disease
AKI	acute kidney injury
JAK/STAT	Janus kinase/signal transducers and activators of transcription
Akt	protein kinase B
ERK	extracellular signal-regulated kinase
PAN	puromycin aminonucleoside
TGF-β1	transforming growth factor beta 1
TRPC6	transient receptor potential cation channel 6
mTOR	mammalian target of rapamycin
ER	endoplasmic reticulum
ASK1	apoptosis signal-regulating kinase 1
MAPKKK	mitogen-activated protein kinase kinase kinase
GAPDH	Glyceraldehyde 3-phosphate dehydrogenase
